# Food parenting stress among caregivers receiving government food assistance: a study from the United States

**DOI:** 10.1016/j.pmedr.2025.103189

**Published:** 2025-07-27

**Authors:** Faith Hardy, Alison Tovar, Emily G. Elenio, Yarisbel Melo Herrera, Michelle Perry, Katherine W. Bauer, Maya K. Vadiveloo

**Affiliations:** a Department of Behavioral and Social Sciences, Brown University School of Public Health, 121 S. Main St., Providence, RI 02906, United States of America; b Friedman School of Nutrition Science and Policy, Tufts University, 150 Harrison Avenue, Boston, MA 02111., United States of America; c Department of Nutritional Sciences, School of Public Health, Michigan University, 1415 Washington Heights, Ann Arbor, MI 48109-2029, United States of America; d Department of Nutrition, University of Rhode Island. 45 Upper College Rd. Kingston, RI 02881, United States of America

**Keywords:** Food parenting stress, Supplemental nutrition assistance program participants, Child diet, Food security, Nutrition education

## Abstract

**Objective:**

Caregivers are expected to implement child feeding recommendations such as providing healthy meals and promoting family meals. However, these expectations may contribute to stress, particularly for families without food security. This study examined food parenting stress and its variation by household food security.

**Methods:**

Baseline data, from Rhode Island and Connecticut (May–September 2023) from an ongoing study assessing the impact of a state-wide incentive program for Supplemental Nutrition Assistance Program participants, were used. Primary caregivers completed an online survey, with socio-demographic questions, household food security, and a Likert-type-scale assessing feeding stressors. Associations between stressors and food security were analyzed using chi-square-tests and multivariable logistic regression.

**Results:**

Among 779 respondents, nearly half of respondents reported that making sure their child eats the right amount of food (46 %), the right kind of food (49 %), and healthy food outside the home (50 %) was ‘moderately’, ‘very’, or ‘extremely’ stressful. Households that did not experience food security had significantly higher odds of reporting stress across all feeding situations vs. those with food security, adjusting for covariates.

**Conclusions:**

Food parenting stress is common and heightened among those that are not food secure. Nutrition education should be paired with supports that address structural barriers.

## Highlights

• Food parenting stress is common among government food assistance recipients.
• Caregivers lacking food security experience greater feeding stress.
• Nutrition education should consider caregivers' food security situations.
• Results suggest pairing nutrition guidance with food access supports.

## 1 Introduction

Consuming a healthy diet during childhood is essential for a child's development and reducing the risk of chronic diseases later in life (Alvez and Alves, 2024). However, most children in the United States do not meet dietary recommendations (Thomson et al., 2019). Caregivers shape children's diet through parenting practices and the home food environment (Mahmood et al., 2021; Patrick and Nicklas, 2005; Savage et al., 2007). Evidence-based recommendations to improve children's diet quality often include providing healthy foods in the home, ensuring access to healthy options outside the home, monitoring food intake, and promoting family meals (Fisher et al., 2021). Little is known however about how caregivers interpret, implement, or cope with them. As caregivers already experience higher levels of stress compared to other adults (American Psychological Association, 2024), trying to follow food parenting recommendations may add to this. This is particularly true for households that are not food secure. Given these challenges, it is important to consider how lack of food security may influence caregivers' stress when trying to implement these guidelines.

Lack of household food security is strongly linked to caregiver stress, anxiety, and depression, which can harm both caregivers and children (Gundersen and Ziliak, 2015). Caregivers in households that are not food secure may find it more difficult to meet both the nutritional and emotional needs of their children (Arlinghaus and Laska, 2021). As caregivers face financial hardships, they may rely on restrictive or pressure-driven feeding strategies to ensure that their children get enough food, consequently impacting their responsiveness to their children's dietary needs (Arlinghaus and Laska, 2021; Baxter et al., 2022; Orr et al., 2020).

Moreover, caregivers that lack food security often receive nutrition education from the Supplemental Nutrition Assistance Program Education (SNAP-Ed) and the Special Supplemental Nutrition Program for Women, Infants, and Children (WIC) (USDA Food and Nutrition Service, 2021; USDA SNAP-Ed Connection, 2024). While these programs play a crucial role in supporting families, certain recommendations may inadvertently contribute to caregiver stress. For instance, advice to avoid forcing children to “clean their plates” may be challenging for caregivers who are not food secure (Arlinghaus and Laska, 2021). In such cases, well-meaning guidance can increase stress, as caregivers may feel that they cannot comply with these suggestions. This, in turn, could further strain their feeding practices and exacerbate existing stress. However, research on how caregivers interpret these recommendations is limited. Identifying whether these recommendations are stressful and if they vary by food security could help tailor nutrition education to reduce pressure and make guidance more feasible.

Therefore, the objectives of this study were to 1) examine the extent to which caregivers from low-income households perceive common food parenting recommendations as stressful and 2) assess how child feeding stress varies by household food security among caregivers participating in the Supplemental Nutrition Assistance Program (SNAP). Understanding this relationship can inform future interventions and programs to ensure that positive food parenting practices are implemented successfully.

## 2 Methods

### 2.1 Study design & participants

The *What's On Your Plate Study* is evaluating Rhode Island's state-wide SNAP incentive program (Eat Well, Be Well) on participants' fruit and vegetable intake and diet quality, with Connecticut SNAP participants serving as a comparison (Vadiveloo et al., 2024). The presented analysis uses cross-sectional baseline data (May–September 2023) from the *What's On Your Plate Kids Study,* a sub-study of the *What's On Your Plate* study. The *What's On Your Plate Kids Study* (Tovar et al., 2025) is assessing the impact of Eat Well, Be Well on parent-reported child outcomes. Study participants had to be at least 18 years of age, be a current SNAP participant, live in Rhode Island or Connecticut, speak English or Spanish, and have access to email and a phone capable of receiving text messages.

Participants were recruited through community partners (predominantly non-profit organizations that work with low-income communities) and text blasts (Elenio et al., 2025). Community partners distributed flyers to facilitate participant sign-ups. Rhode Island and Connecticut WIC offices also sent text message blasts directly through their listservs, which reach all WIC participants who have opted in to receive text messages. Most participants were recruited via text message blasts (59.2 %). An additional 32.6 % were recruited through outreach efforts by community partners using flyers and single-use Quick Response code cards, while 8.1 % were enrolled by research assistants. All participants first completed a brief online screening questionnaire via Qualtrics to confirm eligibility. Participants deemed eligible and unlikely to be a duplicate or bot were directed to complete a survey. Data reported from the survey went through automated data quality checks before participants received incentives, with research assistants conducting additional reviews for suspicious entries. Participants who failed these checks were contacted at least three times by phone for follow-up. Participants who reported in the survey that a child in their household was between the ages of one and eight were then invited via email to complete an additional survey. This study was approved by the Institutional Review Boards (IRBs) at the University of Rhode Island and Brown University and met both institutions' guidelines for the protection of human subjects with regard to safety and privacy.

### 2.2 Participants

Of the 1337 participants who completed the survey for the main *What's on Your Plate* Study, 782 met the eligibility criteria for the *What's on Your Kids Plate* sub-study and completed the caregiver survey. Of those, three were excluded due to ineligibility. A total of 779 participants completed the caregiver survey (379, 48.7 % from Rhode Island and 400, 51.3 % from Connecticut).

### 2.3 Measures

Food Parenting Stress: To develop the food parenting stress items, we drew on existing frameworks and recommendations. Specifically, the items were informed by the fundamental constructs in food parenting practices proposed by Vaughn et al. (Vaughn et al., 2016). In addition, we reviewed feeding recommendations for families with young children published by Healthy Eating Research, a national program of the Robert Wood Johnson Foundation. These resources guided our selection of items that reflect common stressors caregivers face in promoting healthy eating habits within the home. SNAP-Ed materials informed item development but the scale was not formally pilot tested or validated. However, items were reviewed internally for clarity and relevance by members of the research team with expertise in nutrition and early feeding (AT & KB). Questions were designed to broadly capture areas related to food parenting recommendations. Caregivers were asked, “How stressful do you find the following situations?” and provided response options on a 5-point Likert scale (from “Not at all” to “Extremely”): Preparing meals for my child; Making sure my child eats the right kinds of food; Making sure my child eats the right amount of food; Making sure my child doesn't eat too much food; Making sure my child eats healthy food while out of the home, for example at school or childcare; Eating meals together as a family. A total food parenting score was created by summing the individual question values, yielding a range from 6 (no stress) to 30 (high stress). The internal consistency of this total score was high (Cronbach's α = 0.88). To examine variations in caregivers' experiences of child feeding stress across food security levels, we collapsed the overall stress scale and individual stress items into binary outcome variables. High stress was defined as scores at or above the 75th percentile. Consistent with our study objectives, we analyzed the composite stress score as well as each individual item.

Household Food Security: Food security status was assessed using the United States Household Food Security Survey Module: Six-Item Short Form. Participants were categorized as experiencing high/marginal food security (0–1 affirmative responses), low food security (2–4 affirmative responses), and very low food security (5–6 affirmative responses) based on established scoring guidelines. The high/marginal food security category includes households with no or minimal indications of food-access problems or limitations, consistent with the United States Department of Agriculture definitions of food security. Low food security reflects reduced quality, variety, or desirability of diet with little or no indication of reduced food intake, while very low food security reflects disrupted eating patterns and reduced food intake due to limited resources. (Blumberg et al., 1999; U.S. Household Food Security Survey Module: Six-Item Short Form, 2024).

Socio-demographic Characteristics: Participants self-reported this information by completing questions assessing their age, household size, race and ethnicity, educational attainment, employment status, marital status, housing status, length of time using SNAP benefits, self-rated health status, and their child's age. To account for small cell sizes, self-rated health status was collapsed into a binary variable (0 = poor or fair; 1 = good, very good, or excellent).

### 2.4 Statistical analysis

Participants' socio-demographic characteristics and food parenting stress were summarized using univariate statistics. We used chi square tests to determine whether the prevalence of different food parenting stressors and sociodemographic characteristics differed by food security status. Multivariable logistic regression models were used to evaluate the associations between household food security and high parental feeding stress controlling for caregiver level covariates. To isolate the association between household food security and food parenting stress, we adjusted for socio-demographic factors identified in prior literature as potential confounders including caregiver age, education, employment, race, ethnicity, self-rated health status, housing situation, and household size. While our primary objective was not causal inference, we sought to account for variables plausibly associated with both food security and caregiver stress. We applied a consistent adjustment set across models to facilitate comparability and avoid overfitting. Among the variables tested, ultimately race and self-rated health status were retained based on changes in Akaike Information Criterion, odds ratios for food security, the likelihood ratio test, Hosmer-Lemeshow Goodness of Fit test (grouped at 10), and the area under the Receiver Operating Characteristic curve. All statistical analyses were conducted using Stata/SE 16.1, and *p*-values<0.05 were considered statistically significant and all tests were two-tailed.

## 3 Results

The mean age of caregivers was 32 ± 6.62 years, and mean age of their youngest child was 2.4 ± 1.74 years (Table 1). Most respondents identified as women (96 %) and participated in WIC (90 %). Slightly more than one-third were Hispanic (39 %), half identified as White (50 %), and approximately one-fifth as Black/African American (20 %). Slightly less than a third of respondents were categorized as experiencing low household food security (31 %) and 26 % were categorized as experiencing very low household food security (Table 1). No socio-demographic differences by state were observed (data not shown but published previously) (Vadiveloo et al., 2024).

**Table 1:** Self-reported Socio-Demographic Characteristics of 779 Caregivers who Participate in the Supplemental Nutrition Assistance Program from Rhode Island and Connecticut between May–September 2023.

	Overall Sample		Feeding Stress Scale	
	Mean	SD	Mean	SD
Age (years)	32.3	6.6	14	6.3
				
Childs' Age (years)	2.4	1.7		
				
Household Size	4.1	1.5		
				
Gender	Frequency	%		
Man	28	3.6	13.5	6.3
Woman	751	96.4	13.9	6.3
Ethnicity				
Hispaniclat	304	39.0	13.1	6.6
Race				
White	395	50.7	14.1	6.1
Black/African American	158	20.3	15.6	6.8
Multiple Races	69	8.9	13.1	5.6
Other and Unknown	157	20.2	12.5	6.3
Education				
Less than high school graduate or General Education Development	77	9.9	12.7	6.4
High school graduate or General Education Development	280	35.9	14.1	6.3
Some college or trade school	291	37.4	14.1	6.4
College graduate or higher	131	16.8	14.1	6.1
Employment				
Unemployed and not looking for work	225	28.9	13.4	5.9
Unemployed and seeking employment	177	22.7	13.9	6.5
Employed part-time (<30 h/week)	179	23	14.3	6.5
Employed full-time (30+ hours/week)	198	25.4	14.4	6.5
Health Status of primary caregiver				
Poor	27	3.5	17.8	5.6
Fair	252	32.4	14.7	6.3
Good	346	44.4	13.3	6.1
Very good	120	15.4	13.0	6
Excellent	34	4.4	15.4	8.2
Marital Status				
Married/Partnered	252	32.4	13.5	6.1
Divorced/Widowed/Separated	91	11.7	14.4	6.8
Never married	401	51.5	14.2	6.4
Prefer not to answer	35	4.5	13.1	6.1
Housing Status				
Owned Housing	103	13.2	13.3	5.5
Rented or Paid Housing	609	78.2	14	6.4
Friend/Family Housing - No Rent	47	6.0	14.6	6.3
Other (Shelter, Transitional Housing, Unsheltered, other)	20	2.6	15.6	6.3
Length of Time Receiving Supplemental Nutrition Assistance Benefits (SNAP)				
< 1 Month	31	4.0	13.1	6.4
1 Month - 1 Year	129	16.6	13.9	6.1
> Year	581	74.6	14	6.4
I don't know/Prefer not to answer	38	4.9	13.8	6.5
Other Benefits Used				
Special Supplemental Nutrition Program for Women, Infants, and Children (WIC)	699	89.7	13.9	6.3
Medicaid/Medicare	532	68.3	14.1	6.2
Unemployment Benefits	34	4.4	13.5	5.9
Disability Benefits	83	10.7	15.9	5.8
Free/Reduced-Price School Lunch or Breakfast	348	44.7	14.1	6.2
Temporary Assistance for Needy Families (TANF)	69	8.9	13.7	6.3
Food Pantry/Bank/Other that gives/serves food	162	20.8	14.8	5.9
The Child and Adult Care Food Program (CACFP)	69	8.9	14.8	5.7
None	11	1.4	13.2	6.7
Household Food Security				
High/marginal food security	330	42.4	12.7	6.3
Low food security	245	31.5	14.0	6.3
Very low food security	204	26.2	15.9	5.9

SNAP = a federal nutrition program that provides monthly benefits to low-income individuals and families to help them purchase food; WIC = a federal nutrition program providing food and support to low-income pregnant women, new mothers, and young children; TANF = a program offering temporary financial assistance to low-income families; CACFP = a program that reimburses child care providers for serving nutritious meals and snacks.

Household food security was assessed using the U.S. Household Food Security Survey Module: Six-Item Short Form (score range: 0–6). Categories are defined as follows: high/marginal food security = 0–1 affirmative responses; low food security = 2–4; very low food security = 5–6.

Nearly half of respondents reported that making sure their child eats the right amount of food (46 %), the right kind of food (49 %), and healthy food outside the home (50 %) was ‘moderately’, ‘very’, or ‘extremely’ stressful (Table 2). Fewer respondents reported that preparing meals for their child, eating meals together as a family, and making sure their child doesn't eat too much food were ‘moderately’ ‘very’, or ‘extremely’ stressful (34 %, 37 %, and 29 %, respectively). The mean food parenting stress scale score was 14.0 ± 6.3 (Table 2). There were no differences in food parenting stress questions by state (data not shown).

**Table 2:** Food Parenting Stress Situations: Distribution and High vs. Low Stress Categorization among 779 Caregivers who Participate in the Supplemental Nutrition Assistance Program from Rhode Island and Connecticut between May–September 2023.

Food Parenting Stress Situation	Reported Level of Stress n (%)					Cut-offs for Hi-Low Stress		
	Not at All	Slightly	Moderately	Very	Extremely	75 % Cutoff	Low Stress n (%)	High Stress n (%)
Preparing Meals	357 (46)	158 (20)	177 (23)	44 (6)	43 (6)	Moderate or Higher	515 (66)	264 (34)
Right Kind of Food	217 (28)	184 (24)	183 (23)	95 (12)	100 (13)	Very or Higher	584 (75)	195 (25)
Right Amount of Food	240 (31)	180 (23)	165 (21)	105 (13)	89 (11)	Moderate or Higher	420 (54)	359 (46)
Doesn't Eat Too Much Food	421 (54)	134 (17)	117 (15)	60 (8)	47 (6)	Moderate or Higher	555 (71)	224 (29)
Eats Healthy Out of Home	220 (28)	170 (22)	176 (23)	114 (15)	99 (13)	Very or Higher	566 (73)	213 (27)
Eats Meals Together	375 (48)	116 (15)	105 (13)	84 (11)	99 (13)	Moderate or Higher	491 (63)	288 (37)
Combined Food Parenting Stress Scale		Min	Max	Mean	SD			
		6	30	14	6.3	18	557 (72)	222 (29)

In logistic regression models controlling for caregiver race and self-rated health status, very low household food security, compared to high or marginal household food security, was associated with significantly higher odds of reporting high stress in most aspects of child feeding. Caregivers with very low household food security had greater odds of experiencing high stress when preparing meals (AOR = 2.1, 95 % CI: 1.2–3.8), ensuring their child eats the right kind of food (AOR = 2.1, 95 % CI: 1.4–3.1), making sure their child eats the right amount of food (AOR = 2.7, 95 % CI: 1.9–3.8), preventing the child from eating too much (AOR = 1.9, 95 % CI: 1.3–2.8), ensuring the child eats healthy outside the home (AOR = 1.7, 95 % CI: 1.2–2.4), and eating meals together (AOR = 1.9, 95 % CI: 1.3–2.7) (Table 3).

**Table 3:** Adjusted Odds Ratios from Logistic Regression Models between Household Food Security and Food Parenting Stress among 779 Caregivers who Participate in the Supplemental Nutrition Assistance Program from Rhode Island and Connecticut between May–September 2023.

	Preparing Meals (95 %CI)	Right Kind of Food (95 %CI)	Right Amount of Food (95 %CI)	Doesn't Eat Too Much Food (95 %CI)	Eats Healthy Out of Home (95 %CI)	Eats Meals Together (95 %CI)	Combined Food Parenting Stress Scale (95 %CI)
Referent group: High/marginal food security							
Low food security	1.8 (0.99–3.1)	1.5 (0.98–2.2)	1.3 (0.9–1.9)	1.4 (0.97–2.1)	1.5 (1.03–2.2)	1.5 (1.04–2.1)	1.6 (1.1–2.3)
Very low food security	2.1 (1.2–3.8)	2.1 (1.4–3.1)	2.7 (1.9–3.8)	1.9 (1.3–2.8)	1.5 (0.99–2.2)	1.9 (1.3–2.7)	2.2 (1.5–3.3)

Note: All models were adjusted for caregivers' race and binary health status (0 = poor or fair; 1 = good, very good, or excellent).

Household food security was assessed using the U.S. Household Food Security Survey Module: Six-Item Short Form (score range: 0–6). Categories are defined as follows: high/marginal food security = 0–1 affirmative responses; low food security = 2–4; very low food security = 5–6.

In adjusted models, caregivers with low household food security also reported elevated odds of food parenting stress relative to those with high or marginal household food security, although associations were generally smaller and not statistically significant for most domains. Statistically significant associations were observed for stress related to ensuring children eat healthy food outside the home (AOR = 1.5, 95 % CI: 1.03–2.2) and eating meals together (AOR = 1.5, 95 % CI: 1.04–2.1).

Additionally, both very low and low household food security were significantly associated with increased overall food parenting stress in adjusted models. Caregivers with very low household food security had 2.2 times the odds (95 % CI: 1.5–2.8) and low household food security had 1.4 times the odds of high overall stress (95 % CI: 1.02–1.8), compared to those with high or marginal household food security (Table 3).

## 4 Discussion

This study examined food parenting stress and its associations with household food security among caregivers receiving SNAP. We found that food parenting stress is prevalent among this sample, with nearly half of caregivers reporting that certain feeding situations are stressful. We also found that caregivers who reported experiencing both low and very low levels of household food security, compared to households who were food secure, were more likely to report that all recommended food parenting practices explored in this study were stressful. While further research is needed to explore caregiver perceptions, implementing these recommended practices without addressing adequate food access may unnecessarily increase parenting stress.

Our results show that caregivers with low or very low household food security report higher levels of food parenting stress compared to those who are food secure across several different feeding situations, consistent with prior literature (Arlinghaus and Laska, 2021). If families are uncertain about when they will have access to food, it is understandable that, despite knowing certain food parenting practices could benefit their child, these practices may become an additional source of stress. Previous studies have demonstrated that household food insecurity is associated with increased stress and negative mental health outcomes, and that the severity of these outcomes tends to rise as food insecurity worsens(Arlinghaus and Laska, 2021; Berge et al., 2020; Cain et al., 2022). Other evidence suggests that parenting stress and lack of household food security can negatively impact food parenting behaviors and practices (Berge et al., 2023; Orr et al., 2020).

Although the unintended consequences of nutrition education among food insecure families have not been thoroughly studied, if food parenting stress influences feeding behaviors and child diet in ways similar to general parental stress, it could contribute to chronic stress and lead to worsened health outcomes for under-resourced families. While our findings raise important questions about how these recommendations are experienced by families that lack food security, they should not be interpreted as a call to reduce education efforts. Rather, they underscore the importance of considering the context and pairing nutrition education with structural supports—such as food assistance, community-based programs, and responsive policy interventions—that address the root causes of household food security. Alleviating the lack of household food security through these broader efforts is likely to be the most effective and sustainable way to reduce food-related parenting stress. Therefore, rather than withholding nutrition education, it is essential to consider the context and the audience and deliver these recommendations in ways that are sensitive to families' lived experiences and accompanied by the necessary resources and policy solutions to support their implementation.

It is also important to consider that the relationship between household food security, parenting stress, and food parenting practices may be bidirectional (González et al., 2022; Berge et al., 2018). Not only can lack of household food security and stress lead to less supportive or more coercive feeding practices, but the use of controlling feeding strategies can also exacerbate parental stress, creating a feedback loop that perpetuates both stress and suboptimal feeding behaviors. Future research may want to consider also evaluating how whether tailored education and additional supports and inclusion of other stress management techniques affects caregivers and children as food parenting practices are not static and can vary significantly within and across days (Loth et al., 2023) in response to momentary stressors, parental mood, and situational challenges such as time constraints or competing responsibilities.

There are some limitations to this study that are worth noting, first it is cross-sectional, thus, causal relationships cannot be derived. Second, our food parenting stress questions were developed by our research team, based on the prior literature, but have not been validated. This study also only surveyed SNAP participants in Rhode Island and Connecticut, so these results are not generalizable to SNAP-eligible non-participants or participants in other regions. The stress caregivers experience may be influenced by external factors like income, distance to a grocery store, or availability of healthy foods, which vary across the country. This study did not assess associations between food parenting stress and possible outcomes like parenting practices and child diet quality. Future studies should validate the food parenting stress measures and take a longitudinal approach to assess whether food parenting stress is associated with food parenting practices and child diet.

In conclusion, among this large sample of low-income, SNAP participating households, we found that many caregivers reported that trying to implement specific feeding practices was stressful. Our data suggests that caregivers who reported experiencing lack of household food security (vs. those with food security) were more likely to report that all recommended food parenting practices explored in this study were stressful. Other studies have warned against adding more stress on caregivers through well-intentioned education interventions (Parents Under Pressure 2024); however, to our knowledge, this is the first study to assess food parenting stress specifically. Addressing household food security is essential, as improving these conditions could alleviate food parenting stress by tackling the economic factors that contribute to it. Similarly, federal, state, and local policies should prioritize enhancing social support for caregivers, which may also reduce food parenting stress. While evidence-based food parenting recommendations are vital for shaping the diet quality of young children, their effectiveness may be diminished without addressing the underlying social determinants of health.

## CRediT authorship contribution statement

**Faith Hardy:** Writing – original draft, Methodology, Formal analysis, Conceptualization. **Alison Tovar:** Writing – review & editing, Supervision, Resources, Project administration, Methodology, Investigation, Conceptualization. **Emily G. Elenio:** Writing – review & editing, Project administration, Methodology, Investigation, Data curation, Conceptualization. **Yarisbel Melo Herrera:** Writing – review & editing, Resources. **Michelle Perry:** Writing – review & editing, Resources. **Katherine W. Bauer:** Writing – review & editing, Conceptualization. **Maya K. Vadiveloo:** Writing – review & editing, Supervision, Methodology, Funding acquisition.

## Funding

This study was suppported by a grant (2020–85,774) from Bloomberg Philanthropies' Food Policy Program (www.bloomberg.org). The contents of this publication do not necessarily reflect the views or policies of Bloomberg Philanthropies. Bloomberg Philanthropies was not involved in design and conduct of the study; collection, management, analysis, or interpretation of the data; preparation review or approval of the manuscript; or decision to submit the manuscript for publication. Maya Vadiveloo received funding from the K01 Career Development award from the National Heart, Lung, and Blood Institute (5K01HL165104).

## Declaration of competing interest

The authors declare that they have no known competing financial interests or personal relationships that could have appeared to influence the work reported in this paper.

## Data Availability

Data will be made available on request.
